# Frequency of disagreements between producers and veterinarians about pain management in cattle

**DOI:** 10.3168/jdsc.2022-0232

**Published:** 2022-07-22

**Authors:** Sage Mijares, Lily Edwards-Callaway, Elizabeth Johnstone, Lorann Stallones, Noa Román-Muñiz, Catie Cramer, Johann Coetzee

**Affiliations:** 1 Department of Animal Sciences, Colorado State University, College of Agricultural Sciences, Fort Collins 80523; 2 Department of Psychology, Colorado State University, College of Natural Sciences, Fort Collins 80521; 3 Department of Anatomy and Physiology, Kansas State University, College of Veterinary Medicine, Manhattan 66506

## Abstract

Pain management is a key element of ensuring animal welfare. Although the opinions of both producers and veterinarians affect decisions about the use of pain mitigation on cattle operations, little is known about how they communicate about this topic. Given the importance of a veterinary-client-patient relationship for developing pain mitigation protocols, understanding the communication between veterinarians and producers is key to the implementation of robust, industry-wide pain management protocols. The objectives of this survey were to understand how producers and veterinarians may respond to disagreements about pain mitigation and to determine where respondents obtain their knowledge about pain recognition and treatment. Results presented herein are part of a larger study previously described. An online survey was distributed to 6 cattle industry groups. Surveys that were >80% complete were included for analysis (n = 1,066). Approximately half of the respondents identified as producers (497, 46.6%) and half as veterinarians (569, 53.4%). The majority of producers believed that disagreements about the use of pain management in cattle never affected their relationship with their veterinarians (349, 70.2%). The veterinarian respondents indicated more disagreements, although the frequency was relatively low, with 43.9% (250) indicated having a disagreement less than once a year. Most producers and veterinarians indicated they were either “extremely unlikely” or “somewhat unlikely” to dissolve the relationship completely if disagreements about pain management arose (veterinarians: 398/569, 70%; producers: 294/497, 59.1%). Veterinarians and producers reported gaining their knowledge about pain recognition from a variety of sources including personal experience and continuing education opportunities. Disagreements about pain mitigation occurred infrequently; however, this could be due to few discussions about pain management in general. These results indicated that there is opportunity for veterinarians to engage with their producers in more discussions about pain management.

## Highlights

• Cattle producers and veterinarians engage in conversations about pain mitigation.
• Differences in opinion are infrequent and do not appear to affect their relationships.
• Both producers and veterinarians rely on personal experiences to identify pain in cattle.
• There is an opportunity to develop educational programs regarding pain management decisions.

## Graphical Abstract

**Figure:**
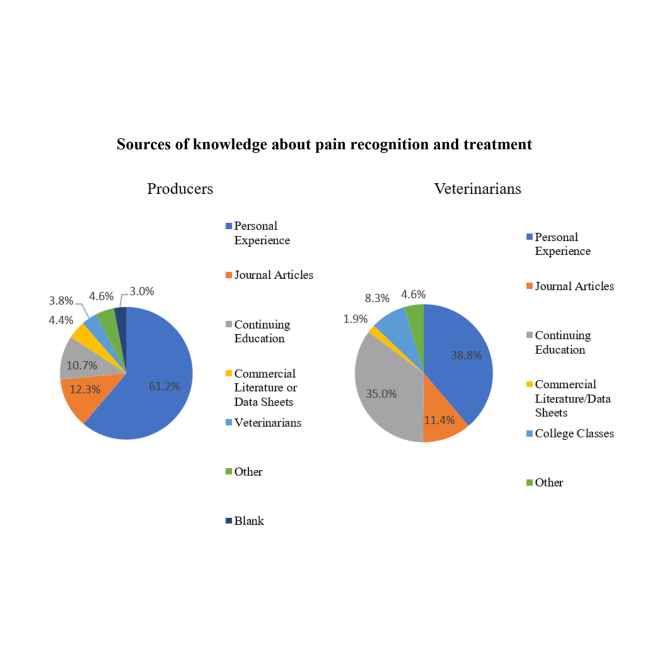
**Summary:** This study explored producer and veterinarian perspectives on pain recognition and pain management for cattle, with the charts above showing sources of knowledge that producers and veterinarians used to gain information about pain recognition and treatment. Although both producers and veterinarians relied heavily on personal experience as their main source of knowledge about pain recognition, both groups identified other sources of information including continuing education opportunities and journal articles.

Pain associated with routine husbandry procedures and other painful conditions continues to be a focus area for the US cattle industry (Whay et al., 2003; Weary et al., 2006; von Keyserlingk et al., 2009). Most of the conversations around pain mitigation focus on management procedures, such as castration and dehorning/disbudding. For example, the American Association of Bovine Practitioners (**AABP**) dehorning guidelines recommend that “pain management be considered the standard of care” for both dehorning and disbudding (AABP, 2019). Additionally, the National Milk Producer's Federation (**NMPF**) Farmers Assuring Responsible Management (**FARM**) Animal Care Program Version 4.0 (FARM, 2020) requires farms to use pain mitigation during dairy calf disbudding. These program manuals outline the importance of conversations with a veterinarian to determine the appropriate pain management plan. Currently, no analgesic drugs have been approved for alleviating pain associated with procedures or conditions other than footrot in the United States. Therefore, in accordance with the requirements set forth in the Animal Medicinal Drug Use Clarification Act (AMDUCA, 1994), extralabel use of analgesic drugs must occur in the context of a valid veterinary-client-patient relationship (**VCPR**). Many different factors affect the use of pain mitigation, but little is known about discussions that occur between producers and veterinarians, which could be a critical factor influencing how frequently pain mitigation is used. The main objective of the study was to enhance understanding of the occurrence of disagreements between producers and veterinarians regarding pain mitigation. Authors hypothesized that although disagreements would be likely to occur, they would be infrequent. An additional objective was to examine how veterinarians and producers obtain knowledge about pain recognition.

This paper presents data not previously reported from a larger survey study described in Johnstone et al. (2021) and Robles et al. (2021). All procedures were approved by the Colorado State University Institutional Review Board (#18–7937H). An online survey was developed using Qualtrics (Provo, UT) and distributed electronically to members of dairy and beef cattle industry and social media groups: FarmProgress master file (n = 34,681), AABP (3,628), Academy of Veterinary Consultants (901), NMPF FARM Evaluators (643), Dairy Moms Facebook group (1,797), and Dairy Girl Network Facebook group (4,927). The survey was made available for 2 mo in summer 2018; 1 to 2 email reminders were sent. The survey included 46 questions (Johnstone et al., 2021). The questions of interest for the present study were Likert scale questions asking about the likelihood of following various courses of actions as a result of disagreement between producers and veterinarians. Questions about where respondents received information about pain recognition and treatment were also included; response options with less than 1% selection were combined into an “other” category for presentation. Demographic questions were also asked. Respondents were asked what their role in the dairy or beef industry was (e.g., veterinarian, producer, or both), and for subsequent questions, branch logic (i.e., creating a custom survey path) was used that was relevant to the role. Only veterinarians and producers were included in the analysis presented here. No identifying information was collected. The only forced-response question was for consent to participate. Surveys were included in analysis if they were >80% complete. Descriptive statistical analyses were performed in Microsoft Excel (Microsoft Corporation).

This survey was distributed to a potential maximum of 46,577 people; 1,790 responses were received for an estimated response rate of 3.8%. After removing incomplete surveys and those from respondents serving both veterinarian and producer roles, a total of 1,066 surveys were included in this analysis. Of the 1,066 responses, 497 (46.6%) identified as producers and 569 (53.4%) identified as veterinarians. Most producers and veterinarians identified as male (399, 80.3% and 361, 63.4%, respectively). Approximately half of producers were 51 to 70 yr old (253, 50.9%), and most veterinarians were under 50 yr old (351, 61.7%).

The majority of producers believed that disagreements about the use of pain management in cattle never affected their relationship with their veterinarians (349, 70.2%; Table 1). Veterinarian respondents indicated that they encountered more disagreements, although the frequency was relatively low; 43.9% (250) indicated having a disagreement about pain management “less than once a year.” Almost a third of veterinarians (158, 27.8%) indicated having disagreements “several times a year,” whereas far fewer producers shared this experience (13, 2.6%). Although infrequent, there were some veterinarians and producers that more commonly experienced disagreement. The majority of producers were “extremely likely” or “somewhat unlikely” to argue with their veterinarian over a disagreement about pain management (337, 67.8%; Table 2). Most veterinarians stated they were “extremely likely” or “somewhat likely” to perform their own research to either support or change their opinion or try to understand the client's wishes (331, 58.2%; Table 3) when disagreements occurred. Most veterinarians were extremely or somewhat unlikely to “terminate the VCPR or relationship with the client” (398, 69.9%). Most producers responded they were either “extremely likely” or “somewhat likely” to “take a chance and try what the veterinarian suggests” (360, 72.4%; Table 2) when disagreements occurred. Many producers were either “extremely likely” or “somewhat likely” to “ask to be provided more information about pain in cattle/perform your own research to either support or change your opinion” (277, 55.7%). Most producers obtained their knowledge about pain recognition and treatment from personal experience (304, 61.2%). Other main sources of knowledge for producers included journal articles (61, 12.3%), continuing education (53, 10.7%), commercial literature or data sheets (22, 4.4%), and veterinarians (19, 3.8%). The majority of veterinarians obtained their knowledge about pain recognition and treatment from personal experience (221, 38.8%) and continuing education (199, 35.0%). Other sources of knowledge for veterinarians included journal articles (65, 11.4%) and college classes (47, 8.3%).

**Table 1:** Frequency of responses to the question, “How often do disagreements about the use of pain management in cattle affect your relationship with the other party?”

Frequency	Veterinarian responses, n (%)	Producer responses, n (%)
Daily	2 (0.4)	0 (0)
Once weekly	4 (0.7)	3 (0.6)
Few times monthly	44 (7.7)	2 (0.4)
Several times a year	158 (27.8)	13 (2.6)
Less than once a year	250 (43.9)	117 (23.5)
Never	108 (19.0)	349 (70.2)
No response	3 (0.5)	13 (2.6)

**Table 2:** Frequency of producer responses to agreement statements related to the question, “If you and your attending veterinarian disagree about the use or lack of use of pain management for your cattle, how likely would you proceed with the following course of action?” (n = 497)

Agreement statement, n (%)	Extremely unlikely	Somewhat unlikely	Neither likely nor unlikely	Somewhat likely	Extremely likely	No response
“Find a different veterinarian who agrees with you”	186 (37.4)	108 (21.7)	121 (24.3)	47 (9.5)	17 (3.4)	18 (3.6)
“Take a chance and try what the veterinarian suggests”	21 (4.2)	43 (8.7)	57 (11.5)	234 (47.1)	126 (25.4)	16 (3.2)
“Argue with veterinarian until they do what you ask”	225 (45.3)	112 (22.5)	96 (19.3)	31 (6.2)	12 (2.4)	21 (4.2)
“Do what you want without the veterinarian knowing”	190 (38.2)	101 (20.3)	105 (21.1)	56 (11.3)	27 (5.4)	18 (3.6)
“Ask to be provided more information about pain in cattle/perform your own research to either support or change your opinion”	42 (8.5)	47 (9.5)	112 (22.5)	179 (36.0)	98 (19.7)	19 (3.8)

**Table 3:** Frequency of veterinarian responses to agreement statements related to the question, “If you and your client disagree about the use or lack of use of pain management for their cattle, how likely would you proceed with the following course of action?” (n = 569)^1^

Agreement statement, n (%)	Extremely unlikely	Somewhat unlikely	Neither likely nor unlikely	Somewhat likely	Extremely likely	No response
“Terminate the VCPR/relationship with the client”	228 (40.1)	170 (29.9)	95 (16.7)	62 (10.9)	9 (1.6)	5 (0.9)
“Do what the client asks”	39 (6.9)	116 (20.4)	161 (28.3)	200 (35.1)	43 (7.6)	10 (1.8)
“Argue with client until they agree with your advice”	101 (17.8)	139 (24.4)	131 (23.0)	162 (28.5)	29 (5.1)	7 (1.2)
“Do what you want and charge the client accordingly”	122 (21.4)	119 (20.9)	103 (18.1)	177 (31.1)	41 (7.2)	7 (1.2)
“Perform your own research to either support or change your opinion or try to understand the client's wishes”	45 (7.9)	66 (11.6)	123 (21.6)	238 (41.8)	93 (16.3)	4 (0.7)

1 VCPR = veterinary-client-patient relationship.

Dairy and beef cattle experience pain during routine procedures such as castration (Molony et al., 1995; Coetzee, 2013; Bergamasco et al., 2021a,b), disbudding or dehorning (Stafford and Mellor, 2005, 2011; Stewart et al., 2008), and branding (Schwartzkopf-Genswein et al., 1997; Tucker at al., 2014, Martin et al., 2022), and from diseases such as lameness (Flower et al., 2008; Coetzee et al., 2017) or mastitis (Leslie and Petersson-Wolfe, 2012; Petersson-Wolfe et al., 2018). Though mitigating pain in cattle for these conditions has the potential to improve animal well-being and production outcomes, pain management implementation is highly variable across procedures and conditions in addition to animal age (Johnstone et al., 2021; Robles et al., 2021). Veterinarians play a key role in managing and promoting animal health on farms. Specifically, in the absence of analgesic drugs approved by the Food and Drug Administration for use alleviating pain associated with most procedures and conditions, a valid VCPR is needed for producers to comply with FARM 4.0 guidelines that require the use of pain mitigation during dairy calf disbudding. Although research is available exploring the perspectives of veterinarians and producers regarding pain mitigation in cattle (Huxley and Whay, 2006; Fajt et al., 2011; Remnant et al., 2017; Johnstone et al., 2021), limited information is available about how the decision to use (or not use) pain mitigation may affect producer-veterinarian relationships. Although many challenges to pain mitigation use have been identified (Johnstone et al., 2021), the way in which veterinarians and producers discuss pain mitigation has not been reported.

Producers and veterinarians had differing opinions on how often disagreements about pain management affected relationships, but the majority of both groups reported that disagreements occurred relatively infrequently; 62.9% (358) and 93.7% (466) of veterinarians and producers, respectively, indicated experiencing a disagreement “less than once a year” or “never.” Compared with producers, fewer veterinarians reported never having a disagreement and perhaps this is related to the nature of the veterinarian's role on the operation; the focus of their visits and conversations with producers is around animal health and welfare and therefore veterinarians potentially have a heightened awareness of topics such as pain management. Although dairy and beef background was not evaluated this could also be a contributing factor to how much pain management is discussed, which would affect the opportunity for differing opinions. Both producers and veterinarians indicated they would seek out information when there was a difference in opinion and generally neither group was likely to take negative action against the other when a disagreement occurred. These results suggest an opportunity for veterinarians to proactively engage in critical conversations about pain mitigation with producers as both parties are open to learning from each other. Exploring how to have these conversations in a constructive way to make improvements in cattle welfare is a needed future area of research. Additionally, based on the data gathered in this study, veterinarians were not the primary source of information for producers on this topic, so finding ways to encourage conversations and increase accessibility of veterinarians is important.

Robles et al. (2021), also reporting data from this survey, indicated that most producers and veterinarians considered their knowledge of recognizing and treating pain in cattle and calves to be adequate. In the current study, most producers reported gaining their knowledge about pain recognition and treatment from personal experience. Robles et al. (2021) found that the oldest age group of producers (>70 yr old) had lower odds of reporting themselves knowledgeable about recognizing and treating pain compared with respondents between 41 and 50 yr of age. This age relationship is contrary to the common belief that older individuals would likely have more life experiences; therefore, understanding details about what type of personal experiences are relevant and meaningful to pain recognition would be helpful. Future research could ask more specific questions about pain recognition to understand what indicators producers and veterinarians rely on to identify pain and assess how accurately they are able to identify pain. Using lameness as an example, some studies have demonstrated that farm managers or owners may underestimate the prevalence of lameness within their herd (Wells et al., 1993; Espejo et al., 2006), suggesting that individual knowledge and experience can influence judgment when assessing painful conditions. Additionally, Dahl-Pedersen et al. (2018) demonstrated moderate agreement at best between producers, veterinarians, and truck drivers in their assessments of lameness and fitness for transport, identifying a need for refinement in definitions and assessment of these conditions.

Veterinarians indicated that they gained their knowledge from personal experience but also cited continuing education. Veterinarians receive formal training on pain identification and management in veterinary school, while producers may not have received comparable formal training. A survey by Lord et al. (2017) of the curriculum content from 21 accredited colleges of veterinary medicine in the United States, Canada, and the Caribbean identified that students received a mean of 15.5 contact hours (ranging from 2 to 40 h) on recognition and management of pain and distress in animals. Lord et al. (2017) did not identify the breakdown of content between small and large animal medicine, which would be important to categorize in future studies. There are opportunities to create continuing education resources for both veterinarians and producers that combine current scientific information about pain management and on-farm practical application. Other studies suggest veterinarians and caretakers are eager for more training on a variety of topics including euthanasia (McGee et al., 2016; Simpson et al., 2020), so it is reasonable to believe that producers and veterinarians would welcome more educational opportunities.

This is one of the first studies exploring the nature of producer-veterinarian conversations regarding pain management for cattle. Results indicate that within the study population veterinarians and producers may have disagreements about pain management but that does not appear to adversely affect relationships. It should be noted that respondents elected to take the survey and therefore may have certain perspectives about pain mitigation, different from individuals who did not participate. There is an opportunity to bring veterinarians and producers together to engage in educational experiences to further understanding of pain mitigation.
